# Creutzfeldt-Jakob Disease With Atypical Magnetic Resonance Imaging Features

**DOI:** 10.7759/cureus.11294

**Published:** 2020-11-02

**Authors:** Muhammad Sohaib Qamar, Amman Yousaf, Anum Nida

**Affiliations:** 1 Internal Medicine, Ozarks Medical Center, West Plains, USA; 2 Radiology, Hamad General Hospital, Doha, QAT; 3 Radiology, Services Institute of Medical Sciences - Services Hospital, Lahore, PAK; 4 Internal Medicine, University at Buffalo - Sisters of Charity Hospital, Buffalo, USA

**Keywords:** creutzfeld-jakob disease, neurodegenerative disease, human prion disease, role of mri, cortical ribboning, basal ganglia high signal intensity, diffusion-weighted images, fluid attenuation inversion recovery, neurological deficit, fatal outcome

## Abstract

Creutzfeld-Jakob disease (CJD) is a rare neurodegenerative condition characterized by rapid progression and fatal outcomes. Patients with progressive dementia and associated atypical features should be investigated, especially with the MRI brain for CJD. Cortical ribboning on diffusion-weighted MRI images is a very crucial diagnostic sign for CJD. Here we present a case of a 52-year-old woman admitted to the hospital after a seizure episode and two-month history of altered mental status. She presented with a 40-minute episode of status epilepticus, necessitating admission to the intensive care unit. Head CT showed no acute intracranial abnormalities, and MRI showed generalized brain atrophy. Electroencephalography (EEG) demonstrated an intermittent slowing of the left hemisphere. Two weeks after admission, she got discharged. Four days later, she presented to the hospital after being found disoriented in a park. MRI showed ventricular dilation and a questionable focus of restricted diffusion in the left thalamus posteriorly. CJD protein panel was collected. Three days after discharge, she was brought to the hospital, and CJD protein testing revealed the presence of 14-3-3 protein, elevated T-tau, and negative real-time quaking-induced conversion (RT-QuIC). The National Prion Disease Surveillance Center reviewed her case, and the CJD diagnosis was confirmed.

## Introduction

Creutzfeldt-Jakob disease (CJD) is a rare, invariably fatal disorder characterized clinically by rapidly progressive neurological decline, myoclonus, and eventual descent into a state of akinetic mutism [1]. The presentation can be variable. It is the most common of the human prion diseases, a group that includes variant CJD, kuru, Gerstmann-Sträussier-Scheinker syndrome, and fatal familial insomnia. The gold standard for CJD diagnosis has traditionally been the presence of classic spongiform changes on brain biopsy. However, in recent years, diagnosis is frequently made based on MRI, electroencephalography (EEG), and laboratory findings alone [2]. MRI sensitivity for sporadic CJD is higher than 90% [3]. In this article, we present a confirmed CJD case in which the clinical presentation was highly suggestive of CJD; however, MRI imaging lacked the typical findings of cortical ribboning or hyperintensity in the basal ganglia.

## Case presentation

A 52-year-old woman with a medical history of stable pulmonary sarcoidosis, hypothyroidism, chronic alcoholic abuse, and protein-calorie malnutrition presented to the hospital with bizarre behavior and altered mental state for two months. She had a normal level of neurological functioning before. She was initially brought to medical attention when she had an episode of a seizure at a rehabilitation facility for an alcohol detoxification program. After admission, she continued to have partial complex seizures with myoclonic extremity movements and one 40-minute episode of status epilepticus, which lead to sedation and intubation in the intensive care unit (ICU). After stepping down to the medical floor, she had a waxing and waning pattern of confusion with visual hallucinations, agitation, and paranoia. However, there were no abnormalities in her interictal neurologic exam. 

**Figure 1:**
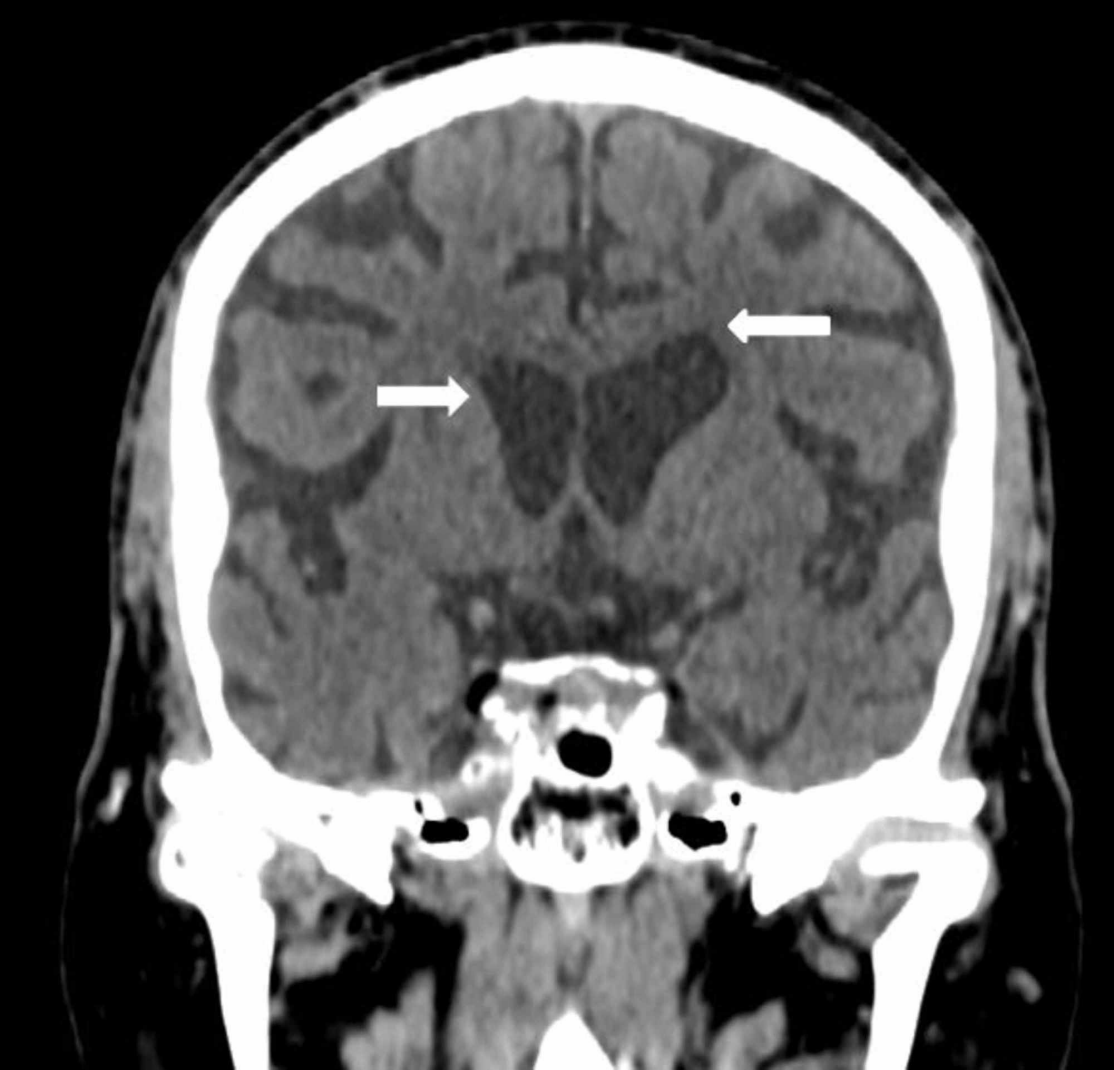
A selected coronal section of the unenhanced CT scan of the head demonstrating prominence of the ventricles (white arrows) and age-related involutional changes

A head CT scan revealed no acute intracranial abnormalities and generalized brain atrophy (Figure 1)

MRI of the brain was also unremarkable. Laboratory work was significant only for mild macrocytic anemia, intermittent hypernatremia, and elevated creatinine kinase of 304 U/L. Her thyroid profile was within normal limits. She was briefly transferred to an outside facility where long-term EEG monitoring showed an intermittent slowing of the left cerebral hemisphere without evidence of spike and wave activity. Levetiracetam was initiated for seizures, and she was discharged to home. 

Four days later, she was brought in by ambulance service to an outside hospital after being found wandering in a park, appearing confused with nonsensical speech. At this time, the physical exam showed a contracted left upper extremity with neglect of right upper limb and bilateral lower limbs. She again experienced profound respiratory distress and required intubation under sedation. A repeated MRI brain showed ventricular dilation with no other significant signal changes. The toxicology screen, antitreponemal antibodies, and HIV testing were negative. Cerebrospinal fluid (CSF) showed red blood cells 81%, neutrophils 56%, glucose 56 mg/dL glucose (normal range 50-80 mg dL or greater than two-thirds of blood glucose), lactic acid dehydrogenase 2.7 U/L (normally it should be less than 40 U/L), protein 28 mg/dL (normal range: 15-45 mg/dL), and myelin basic protein of 6.5 mg/dL (normally it should be less than 4 ng/mL). CSF was negative for cryptococcus, cytomegalovirus, enterovirus, herpes simplex virus 1 and 2, and varicella-zoster virus. Creutzfeldt-Jakob disease (CJD) protein panel was collected at that time, though the results remained pending throughout admission. The differential diagnosis at that time included autoimmune or viral encephalitis, end-stage frontotemporal dementia, Wernicke's encephalopathy, and CJD. Her maintenance therapy in the hospital included levetiracetam, thiamine, and folate with minimal improvement in her mental status for three weeks. Then she was discharged again to the family's care.

**Figure 2:**
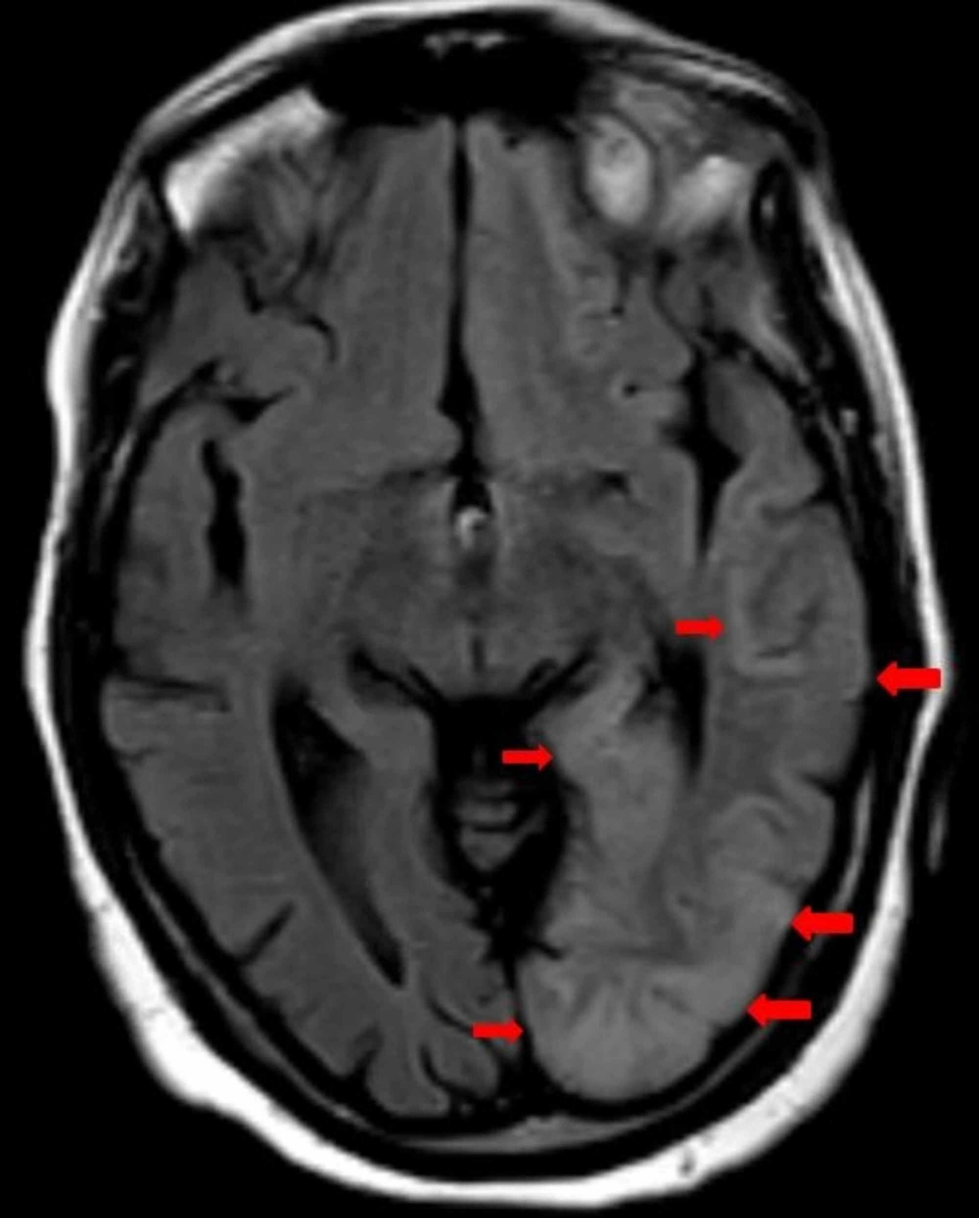
A selected axial T2-weighted FLAIR image It is showing slightly high signal intensity (red arrows) in the left temporal, parietal, and occipital regions. These signals are diffusely involving the white and grey matter. FLAIR: fluid-attenuated inversion recovery.

Three days after the discharge, she again presented to the hospital with myoclonus of the right upper extremity, aphasic, repeating stereotyped phrases, and disorientation. An additional MRI showed high signal intensity involving the left temporal, parietal, and occipital lobes with extension towards the left hippocampus with surrounding edema (Figure 2)

During this admission, CJD protein testing returned and revealed the presence of 14-3-3 protein, elevated T-tau (>4000 pg/mL), and negative real-time quaking-induced conversion (RT-QuIC). National Prion Disease Surveillance Center reviewed and confirmed the diagnosis of Creutzfeldt-Jakob disease. As there is no curative treatment for Creutzfeldt-Jakob disease, comfort care and symptomatic treatment were considered and continued.

## Discussion

Human prion disease occurs at a rate of 1 to 1.5 per 1 million in most developed countries. In the United States, the incidence is around 1.2 in 1 million, with a peak age of onset between 55 to 75 years [4]. Prion diseases can be classified as acquired, hereditary, and sporadic, with sporadic Creutzfeldt-Jakob disease (sCJD) being the most common phenotype, accounting for more than 85% of all human prion disease cases [5]. The disease's main presentation includes rapidly progressive dementia, with behavioral abnormalities, gait ataxia, extrapyramidal features, and eventually, myoclonus. Life expectancy following diagnosis is usually six months to one year [6]. 

CJD's definitive diagnosis is performed through histopathological analysis, which usually shows spongiform brain degeneration, astrocytic gliosis, and neuronal loss due to the accumulation of abnormal prion protein (PrPSc). The spongiform changes refer to grey matter tiny vacuole formation that may combine and give the appearance of holes under the microscope [7]. Detection of PrPSc reactivity with immunohistochemical staining and demonstration of protease-resistant PrPSc showed to have diagnostic value [8]. However, brain biopsies are logistically challenging to perform in these patients due to the need for sterilizing operation rooms and equipment, considering the risk of transmission. Histopathological characteristics can vary depending on the specific molecular phenotype of sCJD, including different clinical presentations and imaging findings [1]. 

Due to CJD's rare incidence, its diagnosis is often not entertained at the time of presentation. Clinical features are usually non-specific and can be confounded with other diseases that present with cognitive impairment, mainly in its early stages. Diagnostic tools include EEG, laboratory findings, and MRI. Early diagnosis is vital to exclude other treatable causes of neurological signals and symptoms presented by patients with suspected sCJD. Professionals can employ different noninvasive modalities to have an accurate and fast diagnosis. EEG is an integral part of sCJD diagnosis and can vary depending on the disease's clinical stage. The most typical finding is periodic sharp wave complexes (PSWC), mainly in the late stages of the disease [9]. However, non-specific findings such as focal or diffuse slowing, as seen in our case, and frontal rhythmic delta activity in the early stages of the disease can be found, despite their limited diagnostic value [10]. 

Laboratory markers of the disease have been discussed widely and matter of controversies. One of the most common markers is protein 14-3-3. Despite previous descriptions of its high sensitivity and specificity, recent literature points towards 14-3-3 being non-specific, as it can be positive in different neurological diseases, including encephalitis and acute stroke [11-13]. Instead, other studies showed that markers such as real-time quaking-induced conversion (RT-QuIC) and Tau protein could be more specific [14,15]. Protein 14-3-3 is still a tool for evaluating patients with suspected sCJD due to more than 85% sensitivity of the test. Protein 14-3-3 and other biomarkers were seen positive mainly in the sporadic form, while more false-negative results are usually seen in genetic and other CJD types [11]. Therefore, physicians should keep in mind the pre-test probability of disease and the results of different tests, including MRI and other lab markers. In our patient, the diagnosis was established based on medical history, clinical presentation, findings of diffusion-weighted (DW) MRI, EEG, and CSF following clinical diagnostic criteria of the World Health Organization [16]. 

Considering the inherent difficulties of performing pathological diagnosis and accessing the accuracy of lab markers, MRI became a vital modality for evaluating patients with suspected prion disease. The abnormal MRI findings have been included in diagnostic guidelines published by the Centers for Disease Control and Prevention (CDC) [17]. Even though early-stage patients can have normal scans, typical findings are usually encountered in patients with sCJD. Diffusion-weighted images (DWI) have been described as the best technique to access sCJD features, and the most common imaging findings are signal hyperintensity in the cerebral cortex and basal ganglia, which can be focal or diffuse [18,19]. Fluid-attenuated inversion recovery (FLAIR) images can also show areas of cortical high-signal intensity and cortical atrophy, as demonstrated in our patient [20]. 

In our patient, 14-3-3 was positive. RT-QuIC was negative; however, the Tau protein was elevated. In the presence of the extremely elevated Tau and the 14-3-3 proteins, her images were reviewed with the National Prion Disease Surveillance Center, who assisted in arriving at the diagnosis. A broad and multidisciplinary approach was required for the correct sCJD diagnosis of the patient.

## Conclusions

Creutzfeldt-Jakob disease (CJD) is one of the rare causes of a rapidly progressive neurological deficit with a short lifespan. This article highlights that patients with progressive dementia and associated atypical features should be investigated, especially with diffusion-weighted and fluid-attenuated inversion recovery (FLAIR) MRI for Creutzfeldt-Jakob disease. Cortical ribboning in the MRI brain is a very crucial diagnostic sign for CJD; however, the absence of it does not rule out CJD, and physicians should investigate the patients with other diagnostic modalities whenever there is a high index of suspicion. This case also redemonstrates that patients have a debilitated and shorter lifespan after the diagnosis, and the unavailability of any treatment warrants the need for further research.

## References

[1] Iwasaki Y (2017). Creutzfeldt-Jakob disease. Neuropathology.

[2] Manix M, Kalakoti P, Henry M, Thakur J, Menger R, Guthikonda B, Nanda A (2015). Creutzfeldt-Jakob disease: updated diagnostic criteria, treatment algorithm, and the utility of brain biopsy. Neurosurg Focus.

[3] Mead S, Rudge P (2017). CJD mimics and chameleons. Pract Neurol.

[4] Collins S, Boyd A, Fletcher A, Gonzales MF, McLean CA, Masters CL (2000). Recent advances in the pre-mortem diagnosis of Creutzfeldt-Jakob disease. J Clin Neurosci.

[5] Prusiner SB (2001). Shattuck lecture--neurodegenerative diseases and prions. N Engl J Med.

[6] Pocchiari M, Puopolo M, Croes EA (2004). Predictors of survival in sporadic Creutzfeldt-Jakob disease and other human transmissible spongiform encephalopathies. Brain.

[7] Ironside JW, Ritchie DL, Head MW (2017). Prion diseases. Handb Clin Neurol.

[8] Hill AF, Joiner S, Wadsworth JD (2003). Molecular classification of sporadic Creutzfeldt-Jakob disease. Brain.

[9] Steinhoff BJ, Räcker S, Herrendorf G (1996). Accuracy and reliability of periodic sharp wave complexes in Creutzfeldt-Jakob disease. Arch Neurol.

[10] Wieser HG, Schindler K, Zumsteg D (2006). EEG in Creutzfeldt-Jakob disease. Clin Neurophysiol.

[11] Sanchez-Juan P, Green A, Ladogana A (2006). CSF tests in the differential diagnosis of Creutzfeldt-Jakob disease. Neurology.

[12] Geschwind MD, Martindale J, Miller D (2003). Challenging the clinical utility of the 14-3-3 protein for the diagnosis of sporadic Creutzfeldt-Jakob disease. Arch Neurol.

[13] Muayqil T, Gronseth G, Camicioli R (2012). Evidence-based guideline: diagnostic accuracy of CSF 14-3-3 protein in sporadic Creutzfeldt-Jakob disease: report of the guideline development subcommittee of the American Academy of Neurology. Neurology.

[14] Park JH, Choi YG, Lee YJ (2016). Real-time quaking-induced conversion analysis for the diagnosis of sporadic Creutzfeldt-Jakob disease in Korea. J Clin Neurol.

[15] Lattanzio F, Abu-Rumeileh S, Franceschini A (2017). Prion-specific and surrogate CSF biomarkers in Creutzfeldt-Jakob disease: diagnostic accuracy in relation to molecular subtypes and analysis of neuropathological correlates of p-tau and Aβ42 levels. Acta Neuropathol.

[16] Gozke E, Erdal N, Unal M (2008). Creutzfeldt-Jacob disease: a case report. Cases J.

[17] (2018). CDC’s diagnostic criteria for Creutzfeldt-Jakob disease, 2018. http://www.cdc.gov/prions/cjd/infection-control.html.

[18] Tschampa HJ, Kallenberg K, Kretzschmar HA, Meissner B, Knauth M, Urbach H, Zerret I (2007). Pattern of cortical changes in sporadic Creutzfeldt-Jakob disease. Am J Neuroradiol.

[19] Meissner B, Kallenberg K, Sanchez-Juan P (2009). MRI lesion profiles in sporadic Creutzfeldt-Jakob disease. Neurology.

[20] Young GS, Geschwind MD, Fischbein NJ (2005). Diffusion-weighted and fluid-attenuated inversion recovery imaging in Creutzfeldt-Jakob disease: high sensitivity and specificity for diagnosis. Am J Neuroradiol.

